# Sarcinaventriculi in association with gastric ulcer: a case report

**DOI:** 10.1099/acmi.0.000550.v3

**Published:** 2024-06-14

**Authors:** Ankita Simkhada, Pritha Acharya, Shreya Shrivastav

**Affiliations:** 1 Department of Pathology, Institute of Medicine, Tribhuwan University Teaching Hospital, Maharajgunj, Kathmandu, Bagmati Province, Nepal

**Keywords:** gram-positive bacteria, gastric ulcer, *Sarcina ventriculi*

## Abstract

*Sarcina ventriculi* is a species of Gram-positive bacteria which has been reported in patients with delayed gastric emptying as well as in association with cases of gastric ulcer and gastric carcinoma. Although it has been reported frequently in veterinary cases as a cause of fatal diseases, the exact pathogenesis in humans has yet to be identified. We report here a case of an elderly male who presented with haematemesis following which an upper gastrointestinal endoscopy was done and a gastric ulcer was revealed. Histopathological examination revealed *S. ventriculi* in association with the ulcer.

## Data Summary

No data were generated or are required for this work to be reproduced.

## Introduction

*Sarcina ventriculi* is a Gram-positive, non-motile, anaerobic coccus-shaped bacterium [1]. It is normally found in soil or water and tends to thrive in a low pH environment [2]. Infection in humans and animals is assumed to occur via consumption of contaminated food. *S. ventriculi* has been reported to be responsible for infection in animals such as livestock, cats and horses, causing gastric dilatation and death, a phenomenon known as ‘abomasal bloating’, but infection in humans is rarely reported [3]. In humans, it was first reported in a patient with gastric pain secondary to bloating and vomiting [4]. It has also been found in the faeces of healthy individuals, particularly vegetarians [5]. *S. ventriculi* is presumed to cause emphysematous gastritis and perforation [6,7]. The specific pathogenesis and diseases caused by this organism in humans have yet to be elucidated. Herein we report a case of *S. ventriculi* infection of the stomach, associated with gastric ulceration, in an 81-year-old male patient.

## Case presentation

**Fig. 1.**
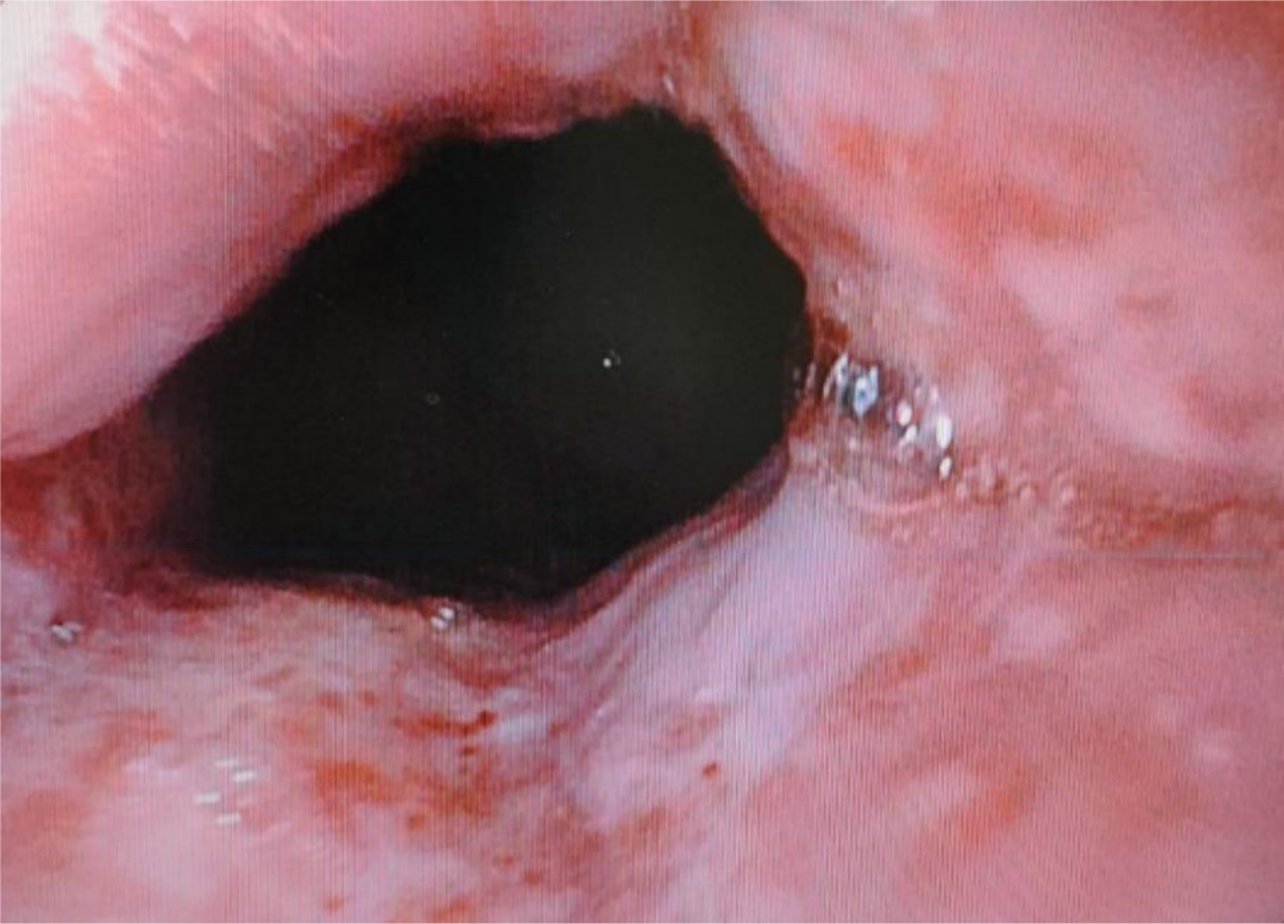
Upper gastrointestinal endoscopy showing Group B reflux oesophagitis.

An 81-year-old man presented to the emergency department with complaints of sudden-onset, severe, abdominal pain and haematemesis for 3 days. He also complained of passage of dark stools. He had no history of chronic disease. He was admitted and routine blood investigations were sent. On admission, his haemoglobin was 6.0 g dl^−1^ for which he received two units of packed red blood cells. He was then scheduled for an upper gastrointestinal endoscopy which showed Grade B reflux oesophagitis (Fig. 1) . The distal oesophagus was dilated and filled with food material. A small area of ulceration was present at the gastro-oesophageal junction. A biopsy was taken from the gastro-oesophageal junction and also random biopsies from the body of the stomach and sent for histopathological examination. Due to his advanced age and symptoms of malignancy, the patient underwent colonoscopy and a computed tomography (CT) scan as a part of routine screening. His colonoscopy reports were unremarkable. A CT scan of the abdomen showed asymmetric thickening of greater curvature of the distal body of the stomach and dilated distal oesophagus, presumed to be due to an inflammatory lesion.

**Fig. 2.**
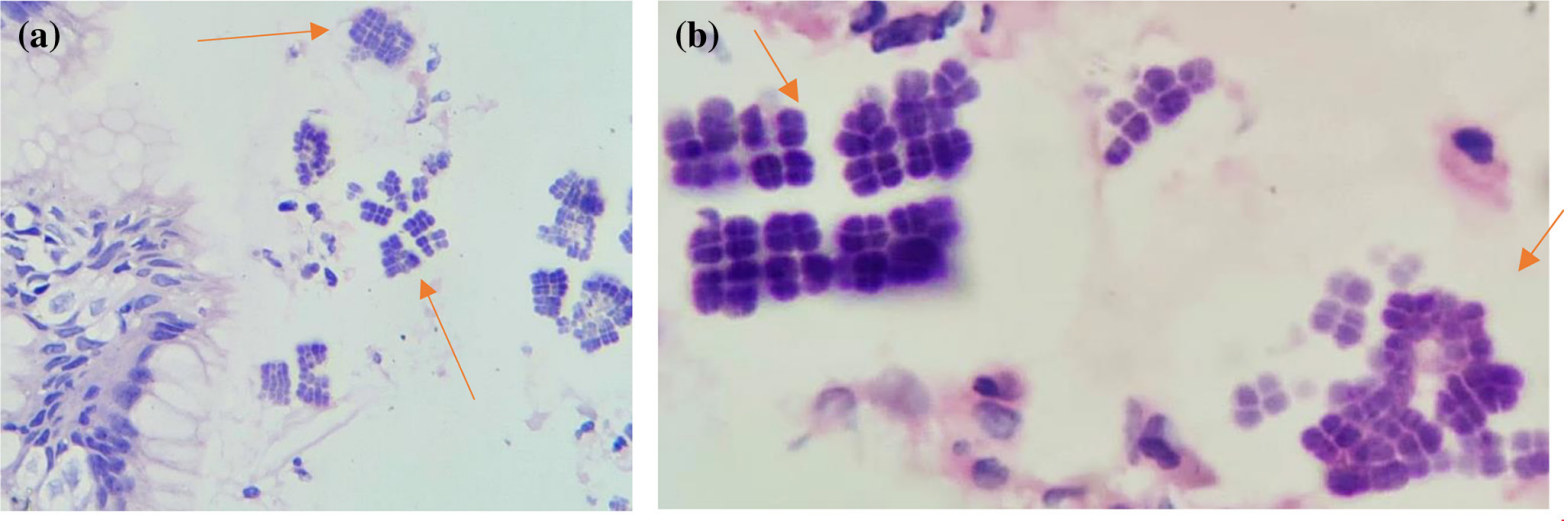
Gastric biopsy showing colonies of *Sarcina ventriculi* arranged in classic octads (red arrows). (a) Haematoxylin and eosin (H&E) stain, 40× magnification; (b) H&E stain, 100× magnification.

Microscopic examination of tissue from the gastro-oesophageal junction revealed oesophageal and gastric mucosal lining with focally ulcerated lining. The lamina propria showed mild acute and chronic inflammatory infiltrates and haemorrhage. Regenerative atypia in the form of nucleomegaly and prominent nucleoli were also seen. In addition, many bacterial colonies arranged in octads, morphologically suggestive of *S. ventriculi*, were noted (Fig. 2). A random biopsy taken from the body of the stomach showed mild chronic active gastritis. No evidence of malignancy was noted in the submitted biopsy. He was discharged on a prescription of proton pump inhibitors and was planned for a repeat endoscopy after 6 weeks.

## Discussion

*S. ventriculi* was documented for the first time in 1842 by John Goodsir in the gastric contents of a patient with stomach pain, bloating and vomiting [4]. Cells of *S. ventriculi* are 1.8–3 µm in diameter and are almost spherical [8]. They occur in tetrads or packets of eight or more and this distinctive morphology is a result of cell division in two planes of growth [9]. Initially, it was assumed to be vegetable material due to its characteristic morphology [4].

The common differential diagnosis of *S. ventriculi* when one encounters an organism with characteristic tetrad packaging on light microscopy is *Micrococcus* species. *Micrococcus* are also Gram-positive cocci, arranged in tetrads with a cell wall that can resemble *S. ventriculi* [10]. However, *Micrococcus* has a considerably smaller cell size, 0.5 µm, and cells tend to form clusters, unlike *Sarcina* species. In addition, *Micrococcus* species are catalase-positive aerobic bacteria whereas *S. ventriculi* is catalase-negative and anaerobic [11]. As such, it is believed that histological features are sufficiently distinctive to identify *S. ventriculi* in a routine haematoxylin and eosin stained slide [8].

Further confirmation can be made using 16S rRNA gene sequence identification by PCR [8]. Another test that allows species confirmation is gene sequencing and detection of homology to the *Sarcina* pyruvate decarboxylase (PDC) gene [8]. The PDC gene product in *S. ventriculi* allows it to convert pyruvate to acetaldehyde and carbon dioxide. This is a unique feature that is not seen in many other bacterial species.

*S. ventriculi* has frequently been mentioned in the veterinary literature as a cause of abomasal bloating and death of livestock [3]. It was first isolated in culture of gastric contents from a human in 1911 when grown under strictly anaerobic conditions [12]. Although a few human diseases associated with *S. ventriculi* have been reported, including cases of emphysematous gastritis and gastric perforation, its role in the pathogenesis of these cases is uncertain [6,7].

Most patients with *S. ventriculi* infection present with clinical symptoms of epigastric pain, nausea, vomiting or dyspepsia [8]. A characteristic finding described in the literature is that of a frothy vomit, also known as ‘sarcinous’ vomit, among infected patients [13]. Endoscopic findings in most cases showed retained food bolus along with features of gastritis, pyloric mass, ulcer or stricture [8].

An extensive review of the literature has shown that most individuals predisposed to infection by *S. ventriculi* have delayed gastric emptying either due to gastroparesis or an outlet obstruction. A variety of causes for gastroparesis such as diabetes, cystic fibrosis, previous bariatric surgeries and gastric outlet obstruction due to mass or stricture have been seen in these cases [14,15]. It has been presumed that these organisms are unlikely to be a cause of these disorders, but rather than being just an incidental finding they can be considered a marker for delayed gastric emptying [8]. Thus, when these organisms are encountered, the cause of delayed gastric emptying should be investigated.

Cases of emphysematous gastritis with a fatal outcome have been reported in the presence of bacterial overgrowth by *S. ventriculi*. Such cases have been reported even in the absence of evidence of delayed gastric emptying [7]. Emphysematous gastritis is a rare entity, with an unclear pathogenesis. It is thought that a pre-existing gastric ulcer acts as a nidus for overgrowth and invasion of organisms into the gastric wall [7]. In such cases, the associated delayed gastric emptying due to any cause favours growth of the organism. Another factor that promotes growth of *S. ventriculi* in such conditions is its ability to survive in extremely low pH conditions [7]. Identifying the symptoms of emphysematous gastritis is important as patients deteriorate rapidly. In addition to non-specific symptoms such as abdominal pain, vomiting and fever, the presence of haematemesis, melena, lactic acidosis and haemodynamic instability are other symptoms, which can help diagnose the condition. Abdominal CT showing air within the gastric wall or portal vein can be helpful for diagnosis and rapid initiation of management [6,7].

Most cases of *S. ventriculi* infection seem to represent an overgrowth of this commensal organism which might not need pharmacological intervention. However, in cases of severe symptoms such as dysphagia or substernal burning, a prescription of proton pump inhibitor combined with prokinetic therapy may be justified [14]. Antibiotic coverage is recommended in cases where the organism is seen in association with ulceration, to minimize the risk of gastric perforation [14].

## Conclusion

The presence of *S. ventriculi* in endoscopic biopsies should be taken as a marker for delayed gastric emptying. As such, diligent search for the underlying cause including malignancy is required. This organism is seldom reported in humans, and due to its rarity, is likely to be overlooked by pathologists who are not familiar with its morphology. Awareness among pathologists is important for correct identification. Further studies to elucidate the pathogenesis of the organism in humans are required.

## Abbreviations

CT: computed tomography
S ventriculi: Sarcina ventriculi
